# Evaluation of Copper and Manganese Concentrations in Commercial Fruit Juices and Nectars Consumed in Brazil by GF AAS

**DOI:** 10.1155/2020/8816068

**Published:** 2020-10-16

**Authors:** Pamela S. M. Rocha, Graziela F. B. Cruz, Ricardo J. Cassella

**Affiliations:** Departamento de Química Analítica, Universidade Federal Fluminense, Outeiro de São João Batista s/n, Centro, Niterói, RJ 24020-141, Brazil

## Abstract

The present work proposes a simple method for direct determination of Cu and Mn in commercial fruit juices and nectars by graphite furnace atomic absorption spectrometry (GF AAS). We analyzed samples of different flavors (orange, mango, passion fruit, peach, and grape) and brands of Brazilian commercial fruit juices and nectars. We also carried out a study to define a suitable temperature program and to optimize the calibration conditions. It was possible to determine Cu and Mn in the samples just after a simple dilution of samples with a 0.70 mol L^−1^ HNO_3_ solution, except in the case of grape juice. We compared the results obtained with the proposed method to those obtained after a traditional treatment based on acid digestion in a microwave oven, and no significant differences were observed (except for grape juice). The accuracy of the method was assessed through a recovery test, which provided recovery percentages in the range of 81–117%. Precision was always better than 8%, and the limits of quantification for Cu and Mn were 6 *μ*g L^−1^ and 9 *μ*g L^−1^, respectively. We analyzed twenty-two samples, and the concentrations of Cu and Mn were in the range of 24.1–321 *μ*g L^−1^ and 116–3296 *μ*g L^−1^, respectively. Statistical analysis using a two-way analysis of variance (ANOVA) at 95% confidence level showed that flavor and brand impacted on the concentration of the analytes in the samples. Among the samples analyzed, the grape juice presented the highest concentrations of both Cu and Mn.

## 1. Introduction

Fruit juices are widely consumed all over the world, especially in tropical countries. According to ABIR (Brazilian Association of Soft Drinks and Non-Alcoholic Beverage Industries), the average consumption of nectar per capita in Brazil increased from 3.9 L in 2010 to 5.3 L per year in 2017 [1]. As the demand for fruit juices increases, it is necessary to develop fast and accurate methodologies to ensure quality and safe products to consumers.

Several studies on fruit juices have been conducted worldwide, especially on the determination of sugar concentration and fruit content. However, it is also necessary to monitor metal concentrations because the ingestion of excessive concentrations of these species can represent a risk for human health [2, 3]. On the other hand, some of them, such as Cu and Mn, are considered essential for the human development. On this basis, the Brazilian Health Regulatory Agency (ANVISA) regulates the minimum amount of minerals and vitamins that must be daily ingested in the form of food, including commercial juices and nectars. The amounts of Cu and Mn recommended by ANVISA are 900 *µ*g and 2.3 mg, respectively [4]. Additionally, in some cases, these elements may have a negative impact on the production process, facilitating redox and precipitation reactions, formation of gels, and even altering the organoleptic characteristics of the juices [5].

Plasma-based atomic spectrometric techniques, such as inductively coupled plasma optical emission spectrometry (ICP OES) and inductively coupled plasma mass spectrometry (ICP-MS), are the most popular analytical techniques used to determine trace metal concentrations in fruit juices [6–13]. On the other hand, the use of atomic absorption techniques such as FAAS and GF AAS is still limited to some specific situations [14–20].

One of the greatest challenges in the determination of trace metals in fruit juices is to deal with possible nonspecific interferences due to the typical high organic carbon content of the samples, which occurs due to the presence of sugars, proteins, and additives. These substances alter the physical characteristics of the samples, leading to the occurrence of strong transport and nebulization interferences. In order to minimize such interferences, most of the published works proposes the application of wet acid digestion [5, 7, 14, 16, 20] or dry ashing [12, 13] approaches to mineralize the carbon present in the samples and simplify their matrices.

Methodologies that aim to reduce the laborious sample pretreatment process have also been reported. In order to eliminate solid interferents present in the medium, some studies centrifuged and/or filtered the juices prior to analyte determination [11, 21, 22]. Direct determination is less usual due to the high viscosity and the complex organic content of the fruit juices [19]. Therefore, the analysis of samples diluted with nitric acid solution has been described as a good alternative to avoid sample digestion. The reported dilutions used in most studies ranged from 2 to 200 times [6, 8, 19, 23].

Only few works were found in the literature on the direct determination of metals in fruit juices using GF AAS. In 1993, Arruda et al. [24] determined aluminum in tomato juice by flow injection analysis coupled with GF AAS. In 2005, Oliveira et al. [25] determined selenium in mango, tomato, and grape juices. Both groups reported the necessity of using chemical modifiers and dilution of samples with 0.2% and 1% (v/v) nitric acid solutions, respectively.

Methods that avoid the previous treatment of the sample are of great interest in the determination of metals in fruit juices, since their use requires a lower time to process the samples, avoids possible losses of the analytes, and reduces the risk of contamination [26]. GF AAS allows a controlled thermal treatment of the samples inside the instrument, making possible the elimination of interferents before the measurement of the analytical signal. Thus, direct determination of metals in fruit juices by GF AAS can be considered a fast and practical alternative to accomplish this task. In this context, the present work proposes a method for the determination of Cu and Mn in nectars and commercial fruit juices consumed in Brazil by GF AAS with a minimum treatment of the samples. Once optimized, the method was applied to the analysis of twenty-two samples of nectars and juices of different flavors and brands commercially available in Brazil, and the impact of these factors on the metal concentrations in the samples was evaluated.

## 2. Materials and Methods

### 2.1. Apparatus

The quantification of the analytes in the solutions was carried out using a Varian AA240Z graphite furnace atomic absorption spectrometer (Mulgrave, Australia), coupled to a GTA-120 atomization unit and a Varian PSD 120 autosampler. The instrument was equipped with a Zeeman-effect background correction system. The graphite tubes with L'vov platform used in this work were longitudinally heated and also obtained from Varian. Individual copper and manganese cathode lamps from Varian were used by applying currents of 3.0 mA and 5.0 mA, respectively. Copper was determined by setting the wavelength at 324.8 nm with a slit width of 0.5 nm, and manganese was measured at 297.5 nm with a 0.2 nm slit. Argon with 99.99% purity (Linde Gases, Macaé, Brazil) was employed as the protective gas. A microwave oven from Berghof (Eningen, Germany), model SpeedWave 4, was employed for sample digestion always using DAK-100 (100 mL) perfluoroalkoxy (PFA) flasks.

### 2.2. Reagents, Solutions, and Samples

All solutions were prepared with deionized water (18.2 MΩ cm^−1^) produced in a Direct Q-3 water purification system (Millipore, Bedford, MA, USA). The concentrated nitric acid used to acidify solutions and samples was of trace metal grade and purchased from Tedia (Fairfield, OH, USA).

Stock solutions (1000 mg L^−1^) of copper and manganese used for the preparation of the standard solutions were both obtained from Tedia (São Paulo, Brazil). The standard solutions of Cu and Mn were prepared daily from dilutions of their respective stock solutions with a 0.70 mol L^−1^ HNO_3_ solution.

Twenty-two samples of commercial fruit juices and nectars, purchased in the city of Rio de Janeiro (Brazil), were analyzed. All beverages were packed in Tetra Pak® packaging. Four different brands of nectars (A, B, C, and D) in five different flavors (orange, mango, passion fruit, peach, and grape) and two brands of fruit juice (A and B) in two different flavors (orange and grape) were evaluated in this work.

### 2.3. Material Decontamination

All materials used in this work were decontaminated through immersion in a nitric acid solution. Polyethylene tubes and glassware were washed with detergent and rinsed with current water, followed by a second rinse with deionized water. Then, they were immersed for 24 h, at least, in a 1.4 mol L^−1^ HNO_3_ solution. Before their use, the materials were rinsed with deionized water and dried at 60°C (except volumetric material).

### 2.4. Direct Determination of Cu and Mn by GF AAS

Direct determination of the analytes by GF AAS in the samples was performed after their appropriate dilution with a 0.70 mol L^−1^ HNO_3_ solution. Two independent aliquots of each sample were analyzed. Integrated absorbance signals were measured by introducing 20 *μ*L of the sample (or standard) solution, previously treated, into the graphite furnace, followed by the application of the temperature program for each metal (Table 1). The measurements were performed in duplicate and without using any chemical modifier in order to avoid the increase in blank signals.

### 2.5. Microwave-Assisted Acid Digestion of Samples

The microwave-assisted acid digestion of the samples was performed in order to have a reference value for the concentrations of Cu and Mn in the samples. For this purpose, 2.0 mL of the samples under the test was mixed with 5.0 mL of concentrated nitric acid directly into the liners, which were then sealed. Afterward, the liners were adjusted to the carrousel of the microwave oven, and the heating program (Table 2) was run. After finishing the heating program, the liners were taken out of the microwave oven cavity and left on the bench until cooling to the laboratory ambient temperature. Then, the liners were opened, their contents were quantitatively transferred to 50 mL volumetric flasks, and the volume was completed to the mark with deionized water. Two independent aliquots of each sample were digested and analyzed.

## 3. Results and Discussion

### 3.1. Optimization of the Temperature Program of GF AAS

The first study was carried out to define a suitable heating program to determine the analytes in the samples by GF AAS. In order to know the thermal behavior of the analytes in the samples, pyrolysis and atomization curves were built up in three different media (aqueous solution, nectar, and juice) for each metal. In this study, we used the samples *S*_2_ (orange nectar, brand B) and *S*_20_ (orange juice, brand B) to construct the pyrolysis and atomization curves. The samples were diluted with a 0.70 mol L^−1^ HNO_3_ solution prior to run the measurements.

**Figure 1:**
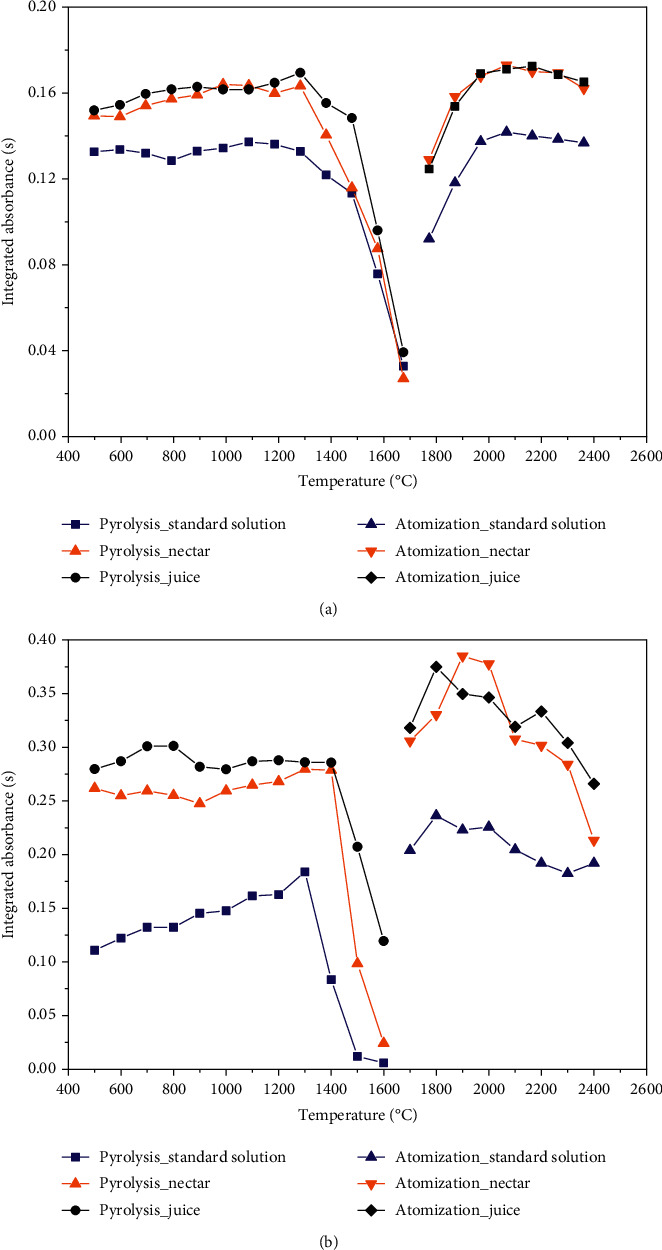
Pyrolysis and atomization curves of (a) Cu and (b) Mn in the two media under test (nectar and juice) and in aqueous standard solution (Cu concentration = 15 *µ*g L^−1^ and Mn concentration = 5.0 *µ*g L^−1^). The curves for nectar and juice were constructed using the samples *S*_2_ and *S*_20_ diluted (1 : 10 to 1 : 40) with 0.70 mol L^−1^ HNO3 solution.

Pyrolysis and atomization curves of Cu and Mn are displayed in Figure 1. The thermal behavior of the two analytes was very similar in the three media, indicating that no important nonspecific interferences affected the measurements. The pyrolysis temperatures for Cu and Mn were set at 1200°C and 1300°C, respectively, in order to ensure total elimination of organic matter without losing the analyte by volatilization. The atomization temperatures were set at 2100°C and 2200°C, for Cu and Mn, respectively, to ensure maximum sensitivity for the measurements in the case of Cu and due to peak shape in the case of Mn. Although we observed lower analytical signals at 2200°C (in comparison with those observed at 2000°C), using this atomization temperature, the Mn peaks were narrower and taller, which improved their differentiation in relation to the baseline.

The temperatures of the drying step were defined to allow total evaporation of the water from the sample matrix or the standard solutions before the pyrolysis step, whereas the cleaning temperature was set at 100°C above the atomization temperature to ensure that any persistent component present in the graphite tube was eliminated. The complete temperature program is given in Table 1.

### 3.2. Evaluation of the Calibration Strategy

Since GF AAS is very sensitive, allowing the quantification of trace amounts of metals, it is possible to work with diluted sample solutions, which can significantly reduce interferences caused by the matrix. Even so, the measurement of the GF AAS analytical signals in juices and nectars are challenging due to the high concentration of sugars. In order to test whether the dilutions were sufficient to eliminate possible nonspecific interferences, analytical curves of each metal were compared to analyte addition curves prepared in different media (nectar or juice, different flavors). These curves were prepared with the samples of five flavors of nectars and the two flavors of juices of brand B.

The comparison between the curves was performed by calculating the slope ratios, which can be defined as the ratios between the slope of analytical curve (S_ac_) and analyte addition curve (S_adc_) for each metal in each different situation. As can be seen in Table 3, the slope ratios for all nectars and orange juice were between 0.82 and 1.18 for both analytes. In these cases, we considered that there was no significant nonspecific interference, since the sensitivity of the analyte addition curves were between 80% and 120% of the sensitivity of the analytical curve. Additionally, Student's *t*-test was applied for the comparison of the slopes of the analyte addition curves with the analytical curve. As can also be seen in Table 3, the values of *t* were always lower than the critical one (*t*_critical_ = 4.30) at 95% confidence level, indicating that there were no differences among the curves. These results demonstrated that the determination of the analytes in the nectars of all flavors and orange juice could be performed using an external calibration approach.

On the other hand, in the case of the samples of grape juice, a significant difference between the sensitivities of the analytical and analyte addition curves for both metals was observed (*t*_values_ of 24.051 for Cu and 15.924 for Mn), with slope ratios of 1.60 and 1.81 for Cu and Mn, respectively. Therefore, for this sample, the direct determination of Cu and Mn by GF AAS could only be possible using an analyte addition approach.

In order to evaluate the results obtained from the slope ratio study, recovery tests were performed for each metal in all samples of brand B. For this purpose, the samples were spiked at four concentration levels (10, 20, 30, and 40 *μ*g L^−1^) with individual Cu and Mn standards. The recovery percentages were calculated at each level as the difference between the concentrations obtained for spiked and nonspiked samples. As expected, the recovery percentages were between 81% and 117% for all nectars and for orange juice, confirming the results obtained in the study of the calibration strategy. Again, grape juice showed a very different behavior. In this case, recovery percentages in the range of 170%–207% were observed, indicating that simple dilution of this type of sample with acid solution was not sufficient to eliminate possible nonspecific interferences. All results obtained in the recovery test are shown in Table 4.

### 3.3. Comparison of Sample Preparation Procedures

**Figure 2:**
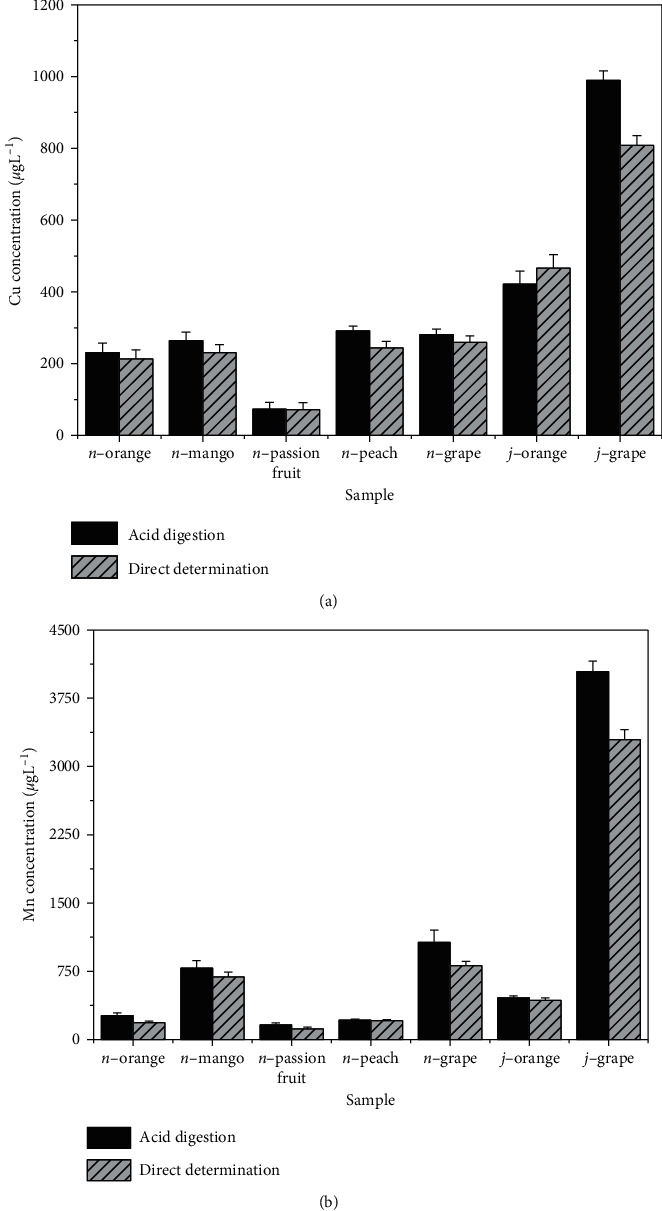
Comparison between the concentrations of (a) Cu and (b) Mn obtained by direct GF AAS determination and after acid digestion of samples. *n* and *j* indicates samples of nectar and juice, respectively.

We also tested the accuracy of the proposed method through the comparison with a reference method. For this, all fruit juice and nectar samples of brand B were submitted to the traditional treatment based on the acid digestion in a microwave oven. The concentrations obtained from the samples treated by acid digestion were compared to the concentrations obtained by direct determination.  Figure 2 shows the results for all samples of nectars (*n*-) and juices (*j*-) of the brand B analyzed by the two procedures. It is important to reinforce that the direct determination of Cu and Mn in the samples was performed using an external calibration strategy with aqueous standard solutions of the analytes, except in the case of grape juice, in which analyte addition curves of Cu and Mn were used instead. In all cases, the samples were diluted with a 0.70 mol L^−1^ HNO_3_ solution to fit in the linear portion of the calibration curves.

In order to confirm whether the results obtained by the two methods were statistically similar, we applied Student's *t*-test for each individual pair of results obtained for Cu and Mn. Before application of Student's *t*-test, each pair of results was evaluated in terms of their variances using the *F*-test. The results are shown in Table 5. As can be seen, all values of *F* were lower than the critical value (*F*_crit_ = 39.00), indicating that, in all cases, the data were homoscedastic. Also, the values of *t* were lower than the critical value (*t*_crit_ = 4.30) and, for this reason, we concluded that no significant difference was observed between the results, evidencing that the simple dilution of the sample with nitric acid solution followed by determination of Cu and Mn by GF AAS can be considered as an effective method for the quantification of metals in these samples.

On the other hand, the determination of both Cu and Mn in the sample of grape juice (also of brand B) presented a different behavior. In these cases, the statistical test showed that the results obtained by acid digestion and direct determination were significantly different at 95% confidence level (*t*_Cu_ = 6.887; *t*_Mn_ = 6.612; *t*_crit_ = 4.30), evidencing that nonspecific interferences cannot be corrected in the grape juice analysis even when using the analyte addition approach as the calibration strategy.

### 3.4. Determination of Analytical Features

After establishing the optimum experimental conditions for the determination of Cu and Mn in the samples, we estimated the analytical features of the method. The instrumental limits of detection (LOD) and quantification (LOQ) were estimated using a calibration curve (10–50 *µ*g L^−1^ for Cu and 2.0–10 *µ*g L^−1^ for Mn) with standard solutions prepared in 0.70 mol L^−1^ HNO_3_ solution and performing ten measurements of the blank solution (0.70 mol L^−1^ HNO_3_). These limits were calculated as LOD = 3.3*σ*/*S* and LOQ = 10*σ*/*S* [27], where *σ* represents the standard deviation of the ten measurements of the blank solution, and *S* represents the sensitivity (slope) of the calibration curve. On this basis, the LOD for Cu and Mn were 2 *µ*g L^−1^ and 3 *µ*g L^−1^, respectively, whereas the LOQ were 6 *µ*g L^−1^ and 9 *µ*g L^−1^, respectively. The typical equations of the calibration curves were *A* = 0.0090 (Cu (*µ*g L^−1^)) + 0.0048 (*r*^2^ = 0.999) and *A* = 0.0347 (Mn (*µ*g L^−1^)) + 0.0026 (*r*^2^ = 0.998). Precision was calculated from five independent determinations of the metals in the sample *S*_1_, and it was always better than 8%.

### 3.5. Application of the Developed Method in the Samples

The concentrations of Cu and Mn in the nectars and orange juices were determined by the method of direct determination by GF AAS, except in the cases of grape juices, in which we determined the analytes after acid digestion of the sample followed by GF AAS determination. The results obtained for Cu and Mn determination are shown in Table 6.

The concentrations of the analytes in orange and grape juices were always higher than in their respective nectars, which can be attributed to the different concentrations of whole juice found in each type of beverage. Grape and orange nectars contain at least 50% of whole juice, whereas the juice itself does not undergo any dilution. Such results may indicate that most metals come from the fruit itself and not from some type of contamination during the production process.

Considering the average concentrations found in the samples according to the flavor, Mn was always found in higher concentrations than Cu, in both nectars and juices. This trend is consistent with the values found in the Brazilian Table of Food Composition [28]. Possibly, the plants absorb more Mn from the soil than Cu, since Mn is vital for the process of energy production during photosynthesis [29, 30].

In order to evaluate whether flavor and brand impact the concentrations of the analytes in the beverages, a two-way ANOVA test was applied for each dataset (Cu and Mn). For both Cu and Mn, the influence of the two factors was considered statistically relevant at 95% confidence level. The test was performed considering the samples with all flavors of nectars, except passion fruit, since it was not possible to purchase passion fruit beverages of all brands.

Despite the ANOVA test indicated that brand impacted the concentration of the analytes, it is not possible to state that the difference observed among the brands is exclusively due to the production process. Several factors may contribute to the variability of metals concentrations in fruits such as soil, climate, seasonality, and others. To establish a safe link between the brand and the analyte concentration in the samples, the entire production process should be monitored and evaluated.

The Cu concentrations determined in all samples were far below the maximum tolerance limit of 10 mg day^−1^ established by the World Health Organization (WHO) [31]. It is also important to highlight that the maximum tolerance limit of Cu in fruit juices is still 30 mg L^−1^, established by an old Brazilian legislation of 1965 [32]. Due to the low toxicity of Mn, both the WHO and national legislation do not set tolerance limits for such metal in this type of beverage.

Given that the metals analyzed are essential to human health, a preliminary comparison between the determined analytes concentration and the Dietary Reference Intakes (DRI) can be performed. As mentioned in the Introduction, DRI for Cu and Mn are 900 *μ*g day^−1^ and 2.3 mg day^−1^, respectively [4]. Therefore, only one cup (200 mL) of grape juice, for instance, would be able to supply 15% and 27% of Cu and Mn DRI, respectively. However, it should be taken into account that the purpose of this work was to determine the total concentration of the metals. So, to have more nutrition information about the analyzed juices, it would be necessary to perform a more detailed study regarding the bioavailability of the metals in the samples.

## 4. Conclusions

The results obtained from the developed method showed that is possible to perform the direct determination of Cu and Mn in most of nectars and fruit juices by GF AAS after minimal pretreatment of the samples by dilution with a 0.70 mol L^−1^ HNO_3_ solution. However, for the grape juice, the acid digestion in a microwave oven was needed, since the nonspecific interferences could not be eliminated even when the optimal conditions for GF AAS measurements were employed.

Twenty-two samples of nectars and juices of different flavors and brands were analyzed. The influence of the flavor and the brand on the concentrations of the analytes in nectars was verified through the two-way analysis of variance (ANOVA), and both factors significantly impacted the concentrations of Cu and Mn in this kind of sample. The grape juice was the beverage that presented the highest concentration of both Cu and Mn. Also, the brands A and B showed the highest concentrations of both analytes.

## Figures and Tables

**Table 1:** Temperature program of the graphite furnace established for the measurement for Cu and Mn.

Step	Temperature (°C)	Ramp (s)	Hold (s)	Ar flow rate (mL min^−1^)
Drying I	95	5.0	—	300
Drying II	120	10.0	14.0	300
Pyrolysis	1200^*∗*^ (Cu), 1300^*∗*^ (Mn)	5.0	4.0	300
Atomization	2100^*∗*^ (Cu), 2200^*∗*^ (Mn)	1.0	3.0	0.0
Cleaning	2200^*∗*^ (Cu), 2300^*∗*^ (Mn)	2.0	—	300

^*∗*^Values established after construction of pyrolysis and atomization curves.

**Table 2:** Heating program employed in the microwave-assisted acid digestion of the samples.

Step	Temperature (°C)	Pressure (bar)	Ramp time (min)	Hold time (min)
1	100	60	1	10
2	200	60	5	10
3	50	60	1	5

**Table 3:** Results obtained in the evaluation of the calibration strategy using samples of different flavors.

Calibration strategy/sample	Type of sample/flavor	Cu^*a*^	Mn^*a*^
Analytical curve	Water	*Y* = (0.0090 ± 0.0002) *x* + (0.0048 ± 0.0055), *r*^2^ = 0.999	*Y* = (0.0347±) *x* + (0.0026 ± 0.0044), *r*^2^ = 0.998
Analyte addition curve/*S*_2_	Nectar/orange	*Y* = (0.0094*x* ± 0.0003) *x* + (0.1347 ± 0.0039), *r*^2^ = 0.999, SR = 1.04, *t* value = 2.495	*Y* = (0.0316±) *x* + (0.3432 ± 0.0184), *r*^2^ = 0.991, SR = 0.91, *t* value = 2.784
Analyte addition curve/*S*_6_	Nectar/mango	*Y* = (0.0106*x* ± 0.0009) *x* + (0.0830 ± 0.0074), *r*^2^ = 0.999, SR = 1.18, *t* value = 4.277	*Y* = (0.0332±) *x* + (0.4046 ± 0.0128), *r*^2^ = 0.997, SR = 0.93, *t* value = 1.653
Analyte addition curve/*S*_9_	Nectar/passion fruit	*Y* = (0.0099*x* ± 0.0005) *x* + (0.0428 ± 0.0022), *r*^2^ = 0.999, SR = 1.10, *t* value = 3.869	*Y* = (0.0310±) *x* + (0.2016 ± 0.0181), *r*^2^ = 0.998, SR = 0.89, *t* value = 2.543
Analyte addition curve/*S*_12_	Nectar/peach	*Y* = (0.0100*x* ± 0.0008)*x* + (0.113 ± 0.0085), *r*^2^ = 0.999, SR = 1.11, *t* value = 3.214	*Y* = (0.0345±) *x* + (0.2512 ± 0.0175), *r*^2^ = 0.995, SR = 0.99, *t* value = 0.149
Analyte addition curve/*S*_16_	Nectar/grape	*Y* = (0.0099*x* ± 0.0008) *x* + (0.0897 ± 0.0076), *r*^2^ = 0.998, SR = 1.10, *t* value = 2.665	*Y* = (0.0293±) *x* + (0.2878 ± 0.0297), *r*^2^ = 0.996, SR = 0.84, *t* value = 3.521
Analyte addition curve/*S*_20_	Juice/orange	*Y* = (0.0087*x* ± 0.0004) *x* + (0.1168 ± 0.0055), *r*^2^ = 0.999, SR = 0.96, *t* value = 1.466	*Y* = (0.0284±) *x* + (0.2353 ± 0.0281), *r*^2^ = 0.999, SR = 0.82, *t* value = 3.816
Analyte addition curve/*S*_22_	Juice/grape	*Y* = (0.0144*x* ± 0.0005) *x* + (0.2910 ± 0.0096), *r*^2^ = 0.993, SR = 1.60, *t* value = 24.051	*Y* = (0.0628±) *x* + (0.1035 ± 0.0062), *r*^2^ = 0.993, SR = 1.81, *t* value = 15.924

^*a*^SR represents the slope ratios and were calculated as S_adc_/S_ac_, where S_ac_ = the sensitivity (slope) of the analytical curve and S_adc_ = sensitivity (slope) of the analyte addition curve. *Y* represents the integrated absorbance (s) and *x* is the concentration of the analyte (*µ*g L^−1^). *t*_value_ was compared to *t*_critical_ = 4.30 (degrees of freedom = 2 at 95% confidence level).

**Table 4:** Results obtained in the recovery tests using samples of brand B.

Sample	Type of sample/flavor	Concentration added (*µ*g L^−1^)	Cu recovery (%)	Mn recovery (%)
*S*_2_	Nectar/orange	10	114	106
		20	109	100
		30	104	99
		40	105	89

*S*_6_	Nectar/mango	10	95	113
		20	102	98
		30	99	93
		40	102	95

*S*_9_	Nectar/passion fruit	10	117	81
		20	106	94
		30	113	95
		40	100	89

*S*_12_	Nectar/peach	10	106	117
		20	108	102
		30	114	105
		40	109	101

*S*_16_	Nectar/grape	10	109	104
		20	113	91
		30	111	88
		40	111	85

*S*_20_	Juice/orange	10	90	88
		20	93	93
		30	95	98
		40	95	93

*S*_22_	Juice/grape	10	175	207
		20	171	201
		30	174	197
		40	170	182

**Table 5:** Statistical evaluation (Student's *t*-test) of the results obtained in the determination of Cu and Mn by the direct GF AAS method and after acid digestion of samples.

Sample	*F* value (Cu)^*a*^	*t* value (Cu)^*b*^	*F* value (Mn)^*a*^	*t* value (Mn)^*a*^
*n*-orange	1.077	0.682	2.966	3.099
*n*-mango	1.162	1.484	2.578	1.378
*n*-passion fruit	1.011	0.075	1.021	2.411
*n*-peach	1.998	3.032	1.677	0.379
*n*-grape	1.358	1.279	7.735	2.764
*j*-orange	1.072	−1.209	1.635	1.166
*j*-grape	1.118	6.887	1.114	6.612

^*a*^F_2,2_ (two-sided test) = 39.00, at 95% confidence level. ^*b*^t_critical_ = 4.30 (degrees of freedom = 2), at 95% confidence level. *n*- and *j*- indicates the samples of nectar and juice, respectively.

**Table 6:** Concentrations of Cu and Mn in the analyzed samples.

Sample	Type	Flavor	Brand	Cu (*µ*g L^−1^)^*b*^	Mn (*µ*g L^−1^)^*b*^
*S*_1_	Nectar	Orange	A	179 ± 5 (20)	249 ± 4 (60)
*S*_2_	Nectar	Orange	B	214 ± 5 (20)	184 ± 4 (20)
*S*_3_	Nectar	Orange	C	24.1 ± 0.8 (3)	246 ± 3 (60)
*S*_4_	Nectar	Orange	D	61.7 ± 4.5 (3)	117 ± 1 (40)
*S*_5_	Nectar	Mango	A	321 ± 12 (20)	2036 ± 2 (500)
*S*_6_	Nectar	Mango	B	231 ± 12 (30)	690 ± 10 (60)
*S*_7_	Nectar	Mango	C	253 ± 3 (20)	814 ± 1 (200)
*S*_8_	Nectar	Mango	D	199 ± 6 (20)	771 ± 2 (200)
*S*_9_	Nectar	Passion fruit	B	72.1 ± 3.8 (20)	116 ± 2 (20)
*S*_10_	Nectar	Passion fruit	C	96.4 ± 1.4 (5)	312 ± 5 (80)
*S*_11_	Nectar	Peach	A	184 ± 4 (20)	246 ± 3 (80)
*S*_12_	Nectar	Peach	B	244 ± 18 (20)	208 ± 1 (20)
*S*_13_	Nectar	Peach	C	183 ± 15 (20)	330 ± 2 (80)
*S*_14_	Nectar	Peach	D	207 ± 5 (20)	247 ± 12 (80)
*S*_15_	Nectar	Grape	A	321 ± 1 (40)	1033 ± 12 (300)
*S*_16_	Nectar	Grape	B	259 ± 3 (30)	812 ± 10 (100)
*S*_17_	Nectar	Grape	C	243 ± 2 (20)	636 ± 18 (100)
*S*_18_	Nectar	Grape	D	606 ± 3 (40)	851 ± 6 (300)
*S*_19_	Juice	Orange	A	215 ± 6 (40)	558 ± 5 (200)
*S*_20_	Juice	Orange	B	466 ± 7 (40)	331 ± 5 (40)
*S*_21_^*a*^	Juice	Grape	A	334 ± 1 (25)	2855 ± 27 (750)
*S*_22_^*a*^	Juice	Grape	B	990 ± 3 (25)	3296 ± 18 (2000)

^*a*^These samples were analyzed after their microwave-assisted acid digestion. ^*b*^Dilution factors applied to the samples are between parentheses. The results (*μ*g L^−1^) are expressed as mean ± standard deviation of two independent determinations.

## Data Availability

The data used to support this study are included within this article.
